# Assessing the TNM Classification for Periprosthetic Joint Infections of the Knee: Predictive Validity for Functional and Subjective Outcomes

**DOI:** 10.3390/jpm15010024

**Published:** 2025-01-10

**Authors:** Arne Kienzle, Sandy Walter, Paul Köhli, Clemens Gwinner, Sebastian Hardt, Michael Müller, Carsten Perka, Stefanie Donner

**Affiliations:** 1 Center for Musculoskeletal Surgery, Clinic for Orthopedics, Charité–Universitätsmedizin Berlin, Corporate Member of Freie Universität Berlin, Humboldt-Universität zu Berlin, and Berlin Institute of Health, 10117 Berlin, Germanypaul.koehli@charite.de (P.K.); clemens.gwinner@charite.de (C.G.); sebastian.hardt@charite.de (S.H.); michael.mueller@mueller-endoprothetik.de (M.M.); carsten.perka@charite.de (C.P.); stefanie.donner@charite.de (S.D.); 2 Julius Wolff Institute and Center for Musculoskeletal Surgery, Charité–Universitätsmedizin Berlin, Corporate Member of Freie Universität Berlin, Humboldt-Universität zu Berlin, and Berlin Institute of Health, 13353 Berlin, Germany; 3 Berlin Institute of Health at Charité-Universitätsmedizin Berlin, BIH Biomedical Innovation Academy, BIH Charité Clinician Scientist Program, 10117 Berlin, Germany; 4 Department of Orthopaedics and Trauma Surgery, Musculoskeletal University Center Munich (MUM), University Hospital, LMU Munich, 80539 Munich, Germany; 5 Zentralklinik Bad Berka GmbH, 99438 Bad Berka, Germany

**Keywords:** periprosthetic joint infection, TNM classification, revision arthroplasty

## Abstract

**Background:** Periprosthetic joint infection (PJI) following knee arthroplasty can significantly compromise patient mobility and quality of life. The newly proposed TNM classification system, adapted from oncology, categorizes PJI severity but has not yet been correlated with both subjective and objective outcomes post PJI treatment. **Objective:** This study evaluates the applicability of the TNM classification system for predicting outcomes in knee PJI revision surgeries. **Methods:** We conducted a retrospective analysis of 108 patients who underwent revision surgeries for knee PJI at our institution from January 2012 to January 2023. We assessed the correlation between the TNM classification and postoperative outcomes using the Knee Society Score (KSS) function and knee score, as well as the Western Ontario and McMaster Universities Osteoarthritis Index (WOMAC). **Results:** The TNM classification demonstrated that higher ‘T’ stages were significantly associated with worse functional and subjective outcomes. The ‘N’ classification had limited predictive value, likely due to treatment adjustments based on pathogen type. The ‘M’ classification correlated with functional outcomes but not with subjective scores, suggesting that patients with more severe preoperative comorbidities might adjust their expectations. **Conclusions:** While the TNM classification shows potential, its current form as a prognostic tool in PJI management is limited. Enhancing the ‘T’ component, coupled with the integration of a validated morbidity score such as the CCI could improve its prognostic value. Despite its shortcomings, the TNM system may still provide valuable prognostic insights for both patients and surgeons in tackling complex PJI.

## 1. Introduction

Periprosthetic joint infection (PJI) is a devastating complication following joint arthroplasty that can significantly impact joint function, mobility, and quality of life [1,2]. The accurate diagnosis and appropriate management of PJI are crucial for achieving successful clinical outcomes. The current gold standard for PJI diagnosis is based on a combination of clinical, laboratory, and radiological analyses [3,4]. Patient-reported postoperative mobility, joint function, and quality of life have been shown to hinge upon various factors including long-term complications, gender, and the complexity of the surgery [5,6,7,8]. However, until recently there was no standardized classification system for grading the severity of PJI. Consequently, there is a paucity of data with which to reliably predict patient outcomes while factoring in disease severity. Despite advances in diagnostic and therapeutic strategies, PJI continues to pose significant challenges due to its heterogeneous clinical presentation and the varying impact of microbial and host factors on outcomes. A unified classification system may not only aid in standardizing treatment but could also enhance the comparability of research findings.

The TNM classification system, developed by the Union for International Cancer Control (UICC), is a widely accepted system for staging malignant tumors. It takes into account the extent of the primary tumor (T), regional lymph node involvement (N), and distant metastases (M) to classify the cancer into different stages [9]. Although developed for cancer staging, this system has been suggested to be used in other diseases such as heart failure [10].

Recently, a new TNM classification system specifically for PJI (Table 1) has been proposed [11,12]. This system considers ‘tissue and implant conditions’ (=‘T’), the presence of resistant organisms (‘non-human cells’ = ‘N’), and pre-existing ‘morbidities’ (=‘M’) to classify the severity of PJI. Previously, a patient’s TNM status has been shown to correlate with several intra-operative parameters including the duration of the explant surgery as well as the amount of blood and bone loss [13]. However, the correlation between this classification system and postoperative patient outcome scores, such as the Western Ontario and McMaster Universities Osteoarthritis Index (WOMAC) and the Knee Society Knee Score (KSS), has not yet been established. Therefore, in this study, we aimed to correlate the new TNM classification for PJI with postoperative patient outcome scores to determine its utility in predicting patient outcomes.

In this paper, we investigate the potential correlation of the new TNM classification for PJI after total knee arthroplasty with patient outcome scores. Specifically, we retrospectively analyzed postoperative KSS and WOMAC scores from a cohort of 64 patients who underwent PJI-dependent revision surgery at our hospital.

## 2. Materials and Methods

### 2.1. Patients

This study received approval from the local ethics board (EA2/083/19), on 3 April 2020 and was conducted following the Declaration of Helsinki. We retrospectively reviewed all patients who underwent total knee arthroplasty revision surgeries due to PJI from 1 January 2012 to 1 January 2023 at our hospital. These patients were cared for in our specialized department using a centralized, interdisciplinary method. A total of 108 patients were included in the study. The inclusion criteria included having a previous total knee arthroplasty and a PJI diagnosis that was treated successfully by the time of discharge. PJI was defined based on the EBJIS criteria [14]: (1) the existence of a sinus tract or purulence around an arthroplasty component; (2) more than 2000 leukocytes/µL or over 70% granulocytes in the synovial fluid; (3) the histology of tissue obtained intra-operatively showing Krenn and Morawietz type II or III [15]; or (4) microbial growth in the synovial fluid, at least two tissue samples (or one sample in cases of high-virulence organisms or antibiotic treatment), or sonication fluid (more than 50 CFU/mL). Only cases classified as ‘confirmed’ were included in this study. Successful treatment at discharge was defined using the modified Delphi criteria [15]: (1) wound healing without fistula, drainage, pain, or recurrent infection; and (2) without PJI-related death due to sepsis.

Patients were excluded from this study if they met any of the following conditions: (1) treatment with DAIR or permanent arthrodesis; (2) no re-implantation of components following implant removal; (3) initial knee arthroplasty performed due to an infection; (4) initial knee arthroplasty or re-implantation performed for trauma without prior signs of aseptic loosening; (5) incomplete postoperative clinical or radiological evaluations; or (6) no follow-up for at least six months post operation. No additional exclusions were applied.

Patient demographics and clinical characteristics are summarized in Table 2. The cohort comprised 108 patients, with a distribution of 48 males and 60 females. The average duration of follow-up was 14.6 months, ranging from 10 to 16 months. The mean Body Mass Index (BMI) was 31.4, ranging from 20.0 to 54.2.

### 2.2. Surgical Technique

Two-stage exchange surgery, recognized as a standard approach for treating chronic PJI, was carried out as described in earlier studies [16]. Initially, this involved the removal of the infected prosthesis, extensive debridement and irrigation, and the insertion of an antibiotic-loaded temporary cement spacer [17,18]. Reimplantation of either a modular or non-modular cemented rotating-hinge prosthesis occurred no sooner than six weeks later, provided there were no clinical or paraclinical signs of infection. All patients were fitted with either a stemmed rotating-hinge or a full-hinged prosthesis, and the procedures were conducted by expert surgeons specializing in PJI management and revision knee arthroplasty. After the reimplantation, patients underwent up to six weeks of antimicrobial treatment. The choice of antibiotics was guided by bacterial susceptibility tests, following Zimmerli’s recommendations [19], and involved consultation with our microbiology specialists.

### 2.3. Follow-Up

To monitor for any complications following revision arthroplasty, our patients were regularly invited to our outpatient clinic. In the first year after surgery, we scheduled patients for radiographic and clinical evaluations every three months. At the one-year follow-up, in addition to the standard assessments, we also performed evaluations using the Knee Society Score (KSS) for knee and function, as well as the Western Ontario and McMaster Universities Osteoarthritis Index (WOMAC) score to assess pain, stiffness, and physical function. TNM scores were determined retrospectively following methodologies previously published [11].

### 2.4. Statistics

Data were analyzed using Excel (v16.30; Microsoft Corporation, Redmond, WS, USA). Where applicable, data were presented as mean or median and analyzed for significance using the Student’s t test. All statistical analyses and plots were performed using R software (R Development Core Team; version: 3.6.3). *p*-values less than 0.05 were considered statistically significant.

## 3. Results

### 3.1. Patient Characteristics

A significant majority (96%) of the patients had multiple comorbidities, with an average Charlson Comorbidity Index (CCI) of 4.4, ranging from 0 to 14. The predominant pathogens identified were Staphylococcus epidermidis, Staphylococcus aureus, and Propionibacterium acnes, together accounting for 60% of all infections. In total, 92 patients underwent two-stage exchange surgery, while 16 patients required multiple-stage exchange surgeries. The average WOMAC score across all patients was 46.5 (range, 6 to 96), while the average KSSs for knee and function were 64.8 (range, 27 to 95) and 60.2 (range, 20 to 100), respectively.

### 3.2. T Score

**Figure 1:**
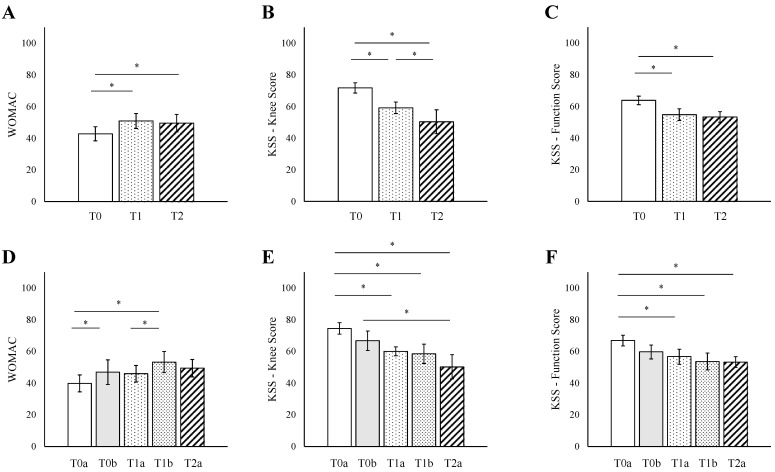
Correlations between TNM ‘T’ Score and Orthopedic Outcomes. (**A**) WOMAC scores versus TNM ‘T’ scores. (**B**) KSS knee scores versus TNM ‘T’ scores. (**C**) KSS function scores versus TNM ‘T’ scores. (**D**) WOMAC scores versus detailed TNM ‘T’ scores. (**E**) KSS knee scores versus detailed TNM ‘T’ scores. (**F**) Bar graph illustrating KSS function scores versus detailed TNM ‘T’ scores. * *p* < 0.05. Scores are presented as means with error bars representing standard deviations. WOMAC—Western Ontario and McMaster Universities Arthritis Index; KSS—Knee Society Score.

The analysis of WOMAC scores revealed a moderate correlation with the TNM stages (Figure 1A); the scores were significantly elevated in patients scored T1 (50.9, range 9 to 94, *p* = 0.04) and T2 (49.5, range 44 to 55, *p* = 0.04) compared to T0 (42.8, range 6 to 96). Correlation analysis found a correlation coefficient (R) of 0.78, although this was not statistically significant (*p* = 0.29). The KSS knee score demonstrated statistically significant differences between T0 and T1 (T0: 71.7, range 50 to 95, T1: 59.1, range 27 to 94, *p* = 0.01) as well as T0 and T2 (T2: 50.3, range 40 to 65, *p* = 0.01), with a strong negative correlation (R = −0.89, *p* = 0.02) with TNM stages (Figure 1B,C). Similarly, for the KSS Function Score, comparisons showed a significant difference between T0 and T1 (T0: 63.8, range 35 to 100, T1: 54.9, range 20 to 90, *p* = 0.02) and between T0 and T2 (T2: 53.3, range 50 to 60, *p* = 0.03), with a strong negative correlation (R = −0.92, *p* = 0.03) with TNM stages.

Further detailed analysis subdividing the T categories for WOMAC scores showed increased scores from T0a (39.9, range 6 to 81) to T0b (47.0, range 6 to 96, *p* = 0.05) as well as T1b (53.3, range 9 to 94, *p* = 0.04) with a correlation coefficient of 0.81 (*p* = 0.12; Figure 1D). Additionally, patients scored T0a (74.5, range 55 to 95) showed significantly higher KSS knee scores compared to those scored T1a (60.0, range 53 to 71, *p* = 0.01), T1b (58.3, range 27 to 94, *p* = 0.01), and T2a (50.3, range 40 to 65, *p* = 0.01; Figure 1E,F). There was a notable negative correlation across subcategories from T0a to T2a with an R value of −0.98 (*p* < 0.001). The KSS function score mirrored this trend, with patients classified as T1a (56.7, range 40 to 80, *p* = 0.02), T1b (53.7, range 20 to 90, *p* = 0.02), and T2a (53.3, range 50 to 60, *p* = 0.03) showing significantly reduced scores compared those scored T0a (66.8, range 40 to 100). Similarly, we found a correlation of −0.84 (*p* = 0.04).

### 3.3. N Score

**Figure 2:**
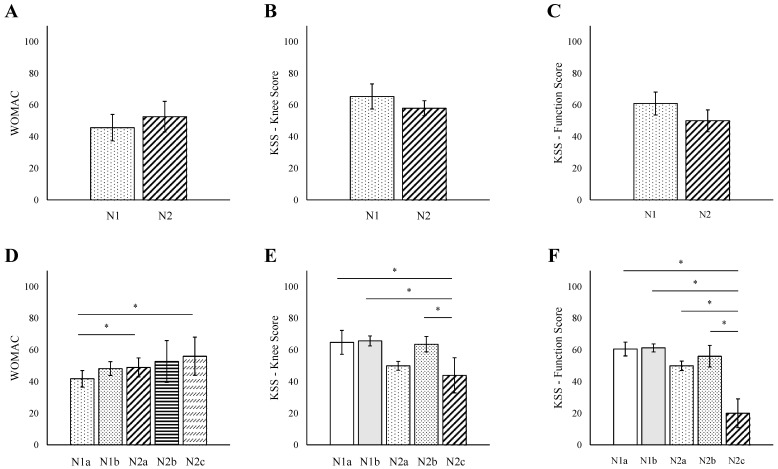
Correlations between TNM ‘N’ Score and Orthopedic Outcomes. (**A**) WOMAC score trends against TNM ‘N’ Scores. (**B**) KSS knee score trends against TNM ‘N’ scores. (**C**) KSS function score trends against TNM ‘N’ scores. (**D**) WOMAC score trends against detailed TNM ‘N’ scores. (**E**) KSS knee score trends against detailed TNM ‘N’ scores. (**F**) KSS function score trends against detailed TNM ‘N’ scores. * *p* < 0.05. Data represented as mean ± SD. WOMAC—Western Ontario and McMaster Universities Arthritis Index; KSS—Knee Society Score.

WOMAC scores showed no N-stage-dependent differences (N1: 45.7, range 6 to 96, N2: 52.6, range 6 to 94, *p* = 0.23; Figure 2A). Similarly, there was no significant difference between N stages for the KSS knee score and KSS function score (Figure 2B,C). However, detailed subdivision analysis showed a significant correlation for WOMAC (R = 0.96, *p* = 0.01), with patients classified as N1a (41.8, range 11 to 87) displaying significantly lower scores compared to those classified as N2a (49.0, range 49 to 89, *p* = 0.04) and N2c (56.0, range 45 to 80, *p* = 0.03; Figure 2D). Conversely, both the KSS knee and KSS function scores depicted a general decline with more advanced N stages, though these were not statistically significant (Figure 2E,F). Of note, the KSS knee and function scores were significantly lower in patients classified as N2c (knee score: 44.0, range 34 to 55, function score: 20.0, range 13 to 40) compared to all other scores besides N2a in the KSS knee score.

### 3.4. M Score

**Figure 3:**
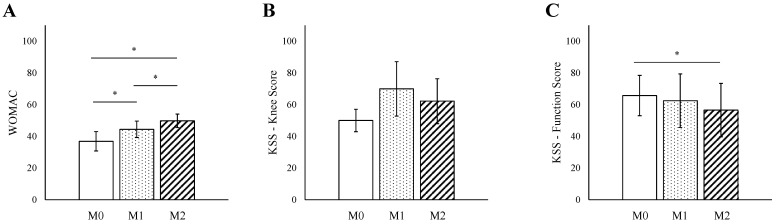
General Correlations between TNM Scores and Outcome Measures Without Detailed Scores. (**A**) General trend of WOMAC scores versus TNM Scores. (**B**) General trend of KSS knee scores versus TNM scores. (**C**) General trend of KSS function scores versus TNM Scores. Displayed data include mean scores and error bars for standard deviations. * *p* < 0.05. WOMAC—Western Ontario and McMaster Universities Arthritis Index; KSS—Knee Society Score.

Furthermore, WOMAC scores showed significant differences with advancing M stages (M0: 36.9, range 13 to 55, vs. M1: 44.4, range 6 to 81, *p* = 0.04, M2: 49.8, range 9 to 96, *p* = 0.03; Figure 3A) and correlated significantly with an R of 0.89 (*p* = 0.04). In contrast, the KSS knee score showed no significant correlation (R = 0.61, *p* = 0.32; Figure 3B). The KSS Function Score, however, was significantly lower in M2 (56.6, range 20 to 90, *p* = 0.04) compared to M0 (65.7, range 50 to 90) but showed no significant correlation (R = −0.48, *p* = 0.10; Figure 3C).

## 4. Discussion

In this study, we investigated the correlation between the newly proposed TNM classification system for periprosthetic joint infections and postoperative patient outcome scores, specifically the WOMAC and KSS knee and function score. While our findings suggest a correlation, the discriminatory power of the TNM classification across different outcomes proved limited, indicating its partial effectiveness.

Previously, several clinical scoring systems have been established for PJI [20,21,22]. These systems have aimed to guide clinical decision making for diagnosis and treatment, to unify scientific communication for the generation of evidence, and to predict treatment results to guide patient counseling. However, each system has its limitations in diagnosing and managing PJI, as well as prediction outcomes. The classification by Schafroth et al. predominantly focuses on the timing of infection onset, classifying PJI as ‘early’, ‘delayed’, and ‘chronic’, yet lacks detail beyond this temporal dimension [20]. Meanwhile, the system proposed by McPherson et al. in 2002 offers another approach to estimate the risk of death or amputation but has not seen widespread adoption. However, while taking the patient’s immune system status into account, it does not consider surgical site conditions, and outcomes like amputation vary greatly, reflecting a lack of consensus on surgical strategies [22,23]. Other classifications, such as the Cierny–Mader system for chronic osteomyelitis, are seldom applied to PJI [21]. The scoring system by Oe et al. evaluates factors such as patient condition, infection duration, prior surgeries, and microbial presence to guide surgical approach selection; however, it lacks broader applicability beyond predicting prosthesis failure [24]. Alternatively, the JS-BACH classification, which considers Joint Specific Bone involvement, Anti-microbial options, Coverage of the soft tissues, and Host status, has proven useful in predicting the likelihood of recurrence and postoperative quality of life in PJI cases [5]. This system grades osteomyelitis or PJI as ‘uncomplicated’, ‘complex’, or ‘with limited treatment options’ and correlates well with clinical outcomes [5,25]. Furthermore, in a retrospective analysis JS-BACH has been shown to significantly correlate with infection recurrence after initial treatment, unlike PJI-TNM, which correlated with the final patient outcome but lacks this specificity [26].

Patient-reported postoperative mobility, joint function, and quality of life have been linked to gender, the complexity of surgery, as well as the occurrence of long-term complications [5,6,7,8]. Recently, the TNM classification has been suggested as a new standard for grading PJI [11,13]. This new classification, proposed by Alt et al. in 2020, uses the principles of the oncological TNM system to categorize the severity of PJI, acknowledging its complexity and the heterogeneity of patient conditions [12]. The TNM classification has also been utilized to enhance PJI therapy by providing a structured framework for treatment stratification [27].

The effective management of PJI hinges upon early detection and treatment, factors not encompassed by the TNM classification. Our findings suggest that the ‘T’ component, which reflects tissue and implant conditions, was the most relevant predictor of both objective and subjective outcomes. However, the TNM system fails to capture the critical conditions of patient presentation such as sepsis, a significant determinant of patient outcomes [28,29], highlighting a major gap in its application and the need to further adapt it; e.g., by adding an “s” prefix. Previous studies have also linked soft and bone tissue defects with worse outcomes—in such cases high-constraint prosthesises can be used with a risk of reduced joint function [30]. Additionally, the number of revision surgeries performed has been shown to be associated with a deterioration in the status of soft and bone tissue, an increased risk for aseptic loosening, and subsequent worse outcomes [8,31].

Conversely, the ‘N’ classification, which accounts for pathogen type, was less predictive of outcomes, likely because treatment strategies were adjusted based on the identified pathogens. Notably, patients classified under ‘N2c’, typically those with fungal infections, showed worse outcomes, aligning with studies indicating that fungal infections often affect a more vulnerable patient demographic and lead to poorer clinical results [6,32].

The ‘M’ score, indicative of existing comorbidities, showed a correlation with functional scores like the WOMAC and KSS function scores. In contrast, the subjective scores were not correlated to the M scoring. Our clinical observations suggest that individuals with objectively poor function often rated their subjective function relatively well. We hypothesize that this is because these patients, having experienced more severe health issues prior to surgery, may have adjusted their expectations and thus perceive their postoperative function to be better than might be anticipated by objective measures. However, the usefulness of another simplified classification for comorbidities is questionable given existing comprehensive classifications like the CCI and the Elixhauser score, which already provide detailed assessments [7,33,34]. Integrating the TNM classification with tools like the CCI could improve its accuracy and provide a clearer assessment of infection severity and host resilience.

This study had several limitations, including a small sample size, varying intervals between surgeries, differences in microbial pathogens, the types of implants used for revisions, and potential variability in documenting the in situ tissue and implant conditions needed for the ‘T’ classification in older cases.

In light of these considerations, while the TNM classification shows potential, its current form as a prognostic tool in PJI management may be considered limited or even redundant. The ‘T’ component was particularly indicative of both objective and subjective outcomes, underscoring the significance of tissue and implant conditions in PJI prognosis. A more detailed subdivision and the development of the ‘T’ component, potentially correlated with an established ‘M’ score like the CCI, could provide a more robust framework for predicting surgical outcomes in PJI. Additionally, the classification should be adjusted to address the timing of treatment in the context of pathogen and biofilm maturation, histological classification, and the presence of recurrent bloodstream infection-causing conditions, such as endocarditis or other implant-associated infections, which complicate the treatment course. Despite its shortcomings, the TNM system may still provide valuable prognostic insights for both patients and surgeons in tackling complex PJI.

## Figures and Tables

**Table 1:** TNM classification proposed by Rupp et al. [11].

T/N/M	Subclassification	Descriptive
T: Tissue and Implant Conditions	0a	Stable standard implant without important soft tissue defect
	0b	Stable revision implant without important soft tissue defect
	1a	Loosened standard implant without important soft tissue defect
	1b	Loosened revision implant without important soft tissue defect
	2a	Severe soft tissue defect with standard implant
	2b	Severe soft tissue defect with revision implant
N: Non-human Cells (Bacteria and Fungi)	0a	No mature biofilm formation (former: acute), directly postoperatively
	0b	No mature biofilm formation (former: acute), late hematogenous
	1a	Mature biofilm formation (former: chronic) without ‘difficult to treat bacteria’
	1b	Mature biofilm formation (former: chronic) with culture-negative infection
	2a	Mature biofilm formation (former: chronic) with ‘difficult to treat bacteria’
	2b	Mature biofilm formation (former: chronic) with polymicrobial infection
	2c	Mature biofilm formation (former: chronic) with fungi
M: Morbidity of the Patient	0	Not or only mildly compromised (Charlson Comorbidity Index: 0–1)
	1	Moderately compromised patient (Charlson Comorbidity Index: 2–3)
	2	Severely compromised patient (Charlson Comorbidity Index: 4–5)
	3a	Patient refuses surgical treatment
	3b	Patient does not benefit from surgical treatment
	3c	Patient does not survive surgical treatment

**Table 2:** Patient Characteristics. BMI = Body Mass Index; CCI = Charlson Comorbidity Index.

Descriptive	Count	Percent	Mean	Range
All patients	108	100.0		
Age [years]			69.1	34.0–85.4
Male	48	44.4		
Female	60	55.6		
Right knee	59	54.6		
Left knee	49	45.4		
Follow-up time [months]			14.6	10.1–14.4
>1 comorbidity	104	96.3		
Clinical scores				
BMI			31.4	20.0–54.2
CCI			4.4	0–14

## Data Availability

Data are available on request from the corresponding author.
